# Using Velocity to Predict the Maximum Dynamic Strength in the Power Clean

**DOI:** 10.3390/sports8090129

**Published:** 2020-09-18

**Authors:** G. Gregory Haff, Amador Garcia-Ramos, Lachlan P. James

**Affiliations:** 1 School of Medical and Health Sciences, Edith Cowan University, Joondalup, WA 6065, Australia; 2 Directorate of Psychology and Sport, University of Salford, Salford, Greater Manchester M5 4WT, UK; 3 Department of Physical Education and Sport, Faculty of Sport Sciences, University of Granada, 18701 Granada, Spain; amagr@ugr.es; 4 Department of Sports Sciences and Physical Conditioning, Faculty of Education, Universidad Católica de la Santísima Concepción, Concepción 4030000, Chile; 5 La Trobe Sport and Exercise Medicine Research Centre (LASEM), Department of Rehabilitation, Nutrition and Sport, School of Allied Health, La Trobe University, Melbourne, VIC 3086, Australia; L.James@latrobe.edu.au

**Keywords:** maximum strength, load-velocity, training intensity, performance tests, resistance training

## Abstract

The primary aim of the present study was to examine the commonly performed training exercise for athlete preparation. Twenty-two recreationally trained males (age: 26.3 ± 4.1 y, height: 1.80 ± 0.07 m; body mass (BM): 87.01 ± 13.75 kg, 1-repetitoon maximum(1-RM)/BM: 0.90 ± 0.19 kg) participated in the present study. All subjects had their 1-RM power clean tested with standard procedures. On a separate testing day, subjects performed three repetitions at 30% and 45%, and two repetitions at 70% and 80% of their 1-RM power clean. During all trials during both sessions, peak velocity (PV) and mean velocity (MV) were measured with the use of a GymAware device. There were no significant differences between the actual and estimated 1-RM power clean (*p* = 0.37, ES = −0.11) when the load-PV profile was utilized. There was a large typical error (TE) present for the load-PV- and load-MV-estimated 1-RM values. Additionally, the raw TE exceeded the smallest worthwhile change for both load-PV and load-MV profile results. Based upon the results of this study, the load-velocity profile is not an acceptable tool for monitoring power clean strength.

## 1. Introduction

Resistance training intensities are commonly prescribed based upon an athlete’s maximum strength levels [1,2], which are typically evaluated with the use of a 1-repetition maximum (1-RM) assessment. While the direct assessment of the 1-RM is well established, safe to perform, reliable and provides a valid method for determining maximal strength [3] several authors have suggested that an alternative method is to estimate the 1-RM with the use of the load-velocity profile [4,5,6]. The ability of the load-velocity profile to predict the 1-RM is based upon the well documented negative relationship between the relative load (i.e., % of 1-RM) and the velocity at which the load is lifted [7,8]. Thus, determining the load-velocity relationship is considered by some to be a useful way for establishing the 1-RM and each of its percentages [9]. Supporters of this method suggest that it is superior to the direct assessment of the 1-RM because it is a non-invasive method that does not interfere with regular training [6,10].

The load-velocity profile has been successfully used to estimate the 1-RM with a variety of exercises including the bench press [10,11], bench-pull [11], seated military press [9] and back squat [2,12]. However, many of the studies that report that the 1-RM can be accurately predicted from the load-velocity relationship have utilized the Smith machine [9,10,11,12]. When free weights have been used to determine the load-velocity relationship for the deadlift [13,14] and the back squat [7], it has been determined that the 1-RM cannot be accurately predicted from velocity measurements. These data imply that there is a possibility that a more accurate estimate of 1-RM can be achieved by creating the load-velocity profile with exercises performed on a Smith machine. This, however, creates an issue for strength and conditioning professionals as the use of free weights is far more commonly used and the load-velocity profile established in the Smith machine may not be transferable to free-weight resistance training activities. 

Recently, Loturco et al. [1] examined the load-velocity profile and the ability to predict the 1-RM with the use of the bench press performed with either free weights or in a Smith machine. While the load-velocity profile was able to accurately predict the 1-RM for the free-weight and Smith machine bench press, there was a significant difference in the 1-RM and mean propulsive velocity at 1-RM between the modes of testing. Specifically, the actual and predicted 1-RM of the Smith machine was found to be 8% higher than the free-weight bench press, whilst the velocity achieved in the Smith machine was ~16% lower than that seen with free weights. Collectively, these differences suggest that the load-velocity profile is exercise mode dependent and that different intensities (i.e., % 1-RM) will be represented by different velocities depending upon if the exercise is performed with free weights or a Smith machine. Importantly, the prediction equations established with each mode of exercise are not interchangeable, suggesting that the load-velocity profile needs to be established on an exercise-specific basis. 

It has also been suggested that exercises which utilize larger muscle groups result in higher movement velocities at the same percentage of 1-RM [11,12]. For example, the velocity at which muscular failure occurs in the Smith machine bench press has been reported to fall between ~0.17–0.18 m·s^−1^, whilst a velocity between 0.31–0.32 m·s^−1^ is associated with the Smith machine back squat [15]. Additionally, it appears that the velocity at which the 1-RM occurs is different depending upon the exercise being examined. For example, Banyard et al. [7] report that the free weight back squat 1-RM occurs at a velocity of 0.24 ± 0.06 m·s^−1^, whilst Lake et al. [14] report that the velocity at 1-RM for the free-weight deadlift occurs at 0.16 ± 0.05 m·s^−1^. Based upon these data, it is clear that to appropriately use movement velocity as a tool for estimating the 1-RM and to prescribe training loads, more exercise-specific load-velocity profiles must be established.

While there has been considerable research establishing the load-velocity profile for the bench press [1,10,11], limited work has explored the free-weight back squat [2,7,12] and deadlift [14]. This is of particular importance as the load-velocity relationship has only been established for a small fraction of exercises that are commonly used in strength and conditioning programs. For example, weightlifting movements (i.e., snatch and clean and jerk) and their derivatives (i.e., pulls, power snatch, power clean, push press, etc.) are commonly employed as part of many strength and conditioning programs [16,17,18] but there are no known studies that have attempted to examine the load-velocity profile in these exercises. Based upon the current available scientific literature, it is likely that the velocity at 1-RM for weightlifting-based exercises, such as the power clean, will be substantially higher than those seen in the bench press, back squat and deadlift because of the fact that the performance of these movements engages a large amount of muscle mass and are performed with free weights. Additionally, similar to the free-weight back squat and deadlift, it is likely that the load-velocity profile of weightlifting movements will not be able to accurately predict the 1-RM. While this line of reasoning is logical, further research is required in order to determine the load-velocity profile of weightlifting movements and their derivatives. Therefore, the primary aim of the present study was to compare the actual power clean 1-RM and the power clean 1-RM predicted from individualized load-velocity profiles. It was hypothesized that the actual and predicted power clean 1-RM would not agree and that the typical error (TE) would be higher than the smallest worthwhile change (SWC).

## 2. Materials and Methods

### 2.1. Experimental Design

Following completion of the instructional sessions, all subjects were evaluated for 1-RM power clean strength, which represented the criterion condition. Peak velocity (PV) of the successful 1-RM attempt was acquired with the use of a GymAware (GYM) (version 5.1, Kinetic Performance Technologies, Canberra, Australia) and used for subsequent analyses. Peak velocity was selected as the primary experimental condition (PV-C) due to its close association with performance in power-based exercises and the recommendation that this velocity measure may be more appropriate for analyzing multi-joint ballistic movements [19], like the power clean. Between four and seven days later, subjects returned to the laboratory and performed the power clean with loads of 30, 45, 70 and 80% 1-RM while PV measures were simultaneously recorded. The resulting load-velocity relationship was then used to calculate individualized linear regression equations in order to predict a 1-RM value using each participant’s known 1-RM velocity. The validity of the predicted 1-RM with reference to the criterion value was then investigated. 

Additionally, a median split of the group on the basis of 1-RM power clean strength was performed followed by the aforementioned analysis to investigate the influence of power clean ability on the validity of the predicted value. A second experimental condition (MV-C) was conducted on a subsample of subjects (*n* = 19) using the mean velocity (MV) in the load-velocity relationship, which was then related to the actual 1-RM achieved in the power clean.

### 2.2. Subjects

Twenty-two recreationally trained males (age = 26.3 ± 4.1 y, height = 1.80 ± 0.07 m, body mass (BM) = 87.01 ± 13.75 kg, training experience = 2.30 ± 1.39 y) who could proficiently perform the power clean (power clean 1-RM/BM = 0.90 ± 0.19 kg) and were free of musculoskeletal injury took part in this investigation. Eligibility required all subjects receive three one-hour instructional sessions on power clean technique delivered by a strength and conditioning professional accredited with the Australian Strength and Conditioning Association and certified with the National Strength and Conditioning Association (l). Subjects exhibiting a range of power clean strength levels were included in the present study in order to allow comparison between stronger and weaker individuals, based upon a median split on the basis of maxima 1-RM power clean strength. All subjects provided their written informed consent and the investigation was approved by the Bellberry Human Research Ethics Committee (2016-04-269). 

### 2.3. Power Clean 1-RM 

The criterion testing session was initiated with a standardized dynamic warm-up consisting of squatting and lunging movements, in addition to the performance of countermovement jumps at progressively increasing intensities. Following the completion of the warm-up, a power clean protocol consisting of three repetitions at 30% and 50% of the estimated 1-RM and one repetition at 70% and 90% of the estimated 1-RM were completed. After the completion of the 90% trial, the load was increased by 2.5 kg increments until a 1-RM was achieved, with a maximum of five 1-RM attempts given [20]. A second attempt at a failed load was allowed to ensure a true maximal effort was attained. Two minutes of passive recovery was allotted between warm-up sets, while a 3 to 5 min rest interval was provided between 1-RM power clean attempts. A lift was considered successful if the participant received the bar no deeper than an internal knee angle of 90 degrees, as visually assessed by the primary investigator. PV and MV were collected for each 1-RM effort with the use of a GYM and the value attained from the 1-RM used for analysis. 

### 2.4. Predicted Power Clean 1-RM

Four to seven days following the initial testing session, subjects returned for the assessment of velocities at four incremental, predetermined loads. The session began with an identical dynamic warm-up to that of the criterion session, after which the subjects performed three repetitions of the power clean at 30 and 45%, and two repetitions at 70 and 80% of the 1-RM power clean load established in the criterion session. Repetitions were performed non-continuously (the performer was required to ‘reset’ between repetitions) and with maximal intent. Velocities were recorded for each loading condition, with the repetition producing the highest respective peak or mean velocity used for all analyses. 

### 2.5. Instrumentation 

The GYM was placed in a position that was perpendicular to the right collar of the barbell and the cable was attached 100 mm immediately proximal to the right collar of the barbell using a Velcro strap. The GYM recorded the displacement–time curve data by determining the changes in barbell position and used a sensor that determined the angle that the cable leaves the unit in order to correct for any horizontal plane [21]. The device sampled and timestamped the barbell displacement data at 20-millisecond time points and down-sampled to 50 Hz for analyses [22]. Velocity data were calculated from the first derivative of the change in barbell position with respect to time. The GYM device was selected for velocity analysis because it has been consistently shown to produce reliable data with loads between 40–90% when used to examine the velocities achieved during multi-joint free-weight exercises [7,13]. 

### 2.6. Statistical analysis

**Figure 1:**
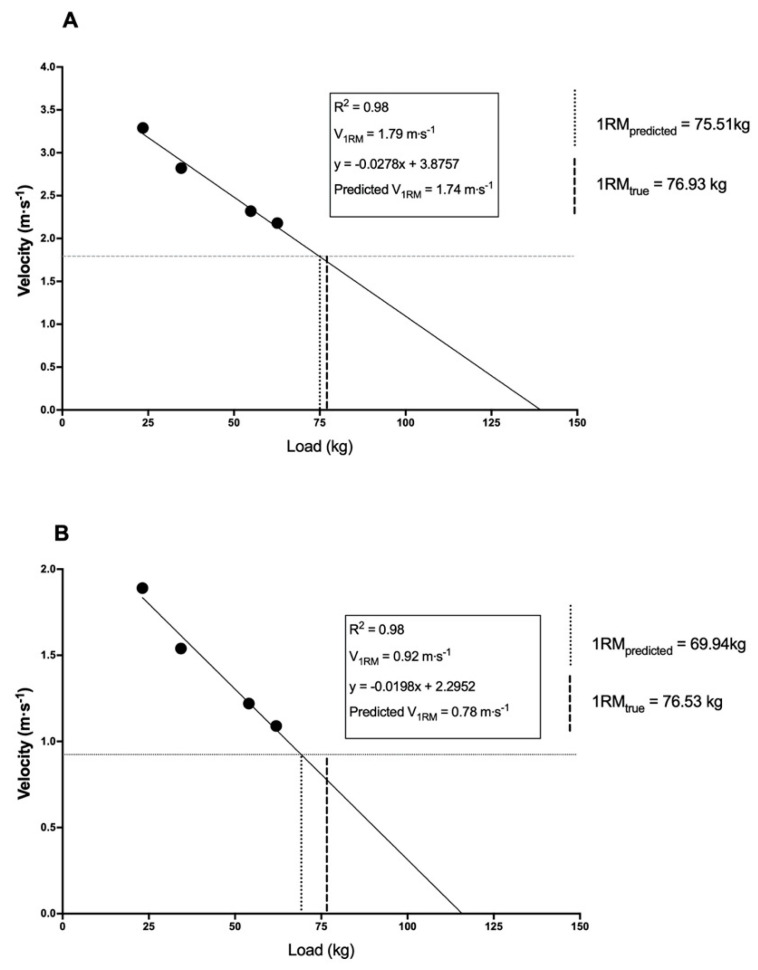
Predicted 1-Repetition Maximum (1-RM) from the peak (**A**) and mean (**B**) velocity attained for 30, 45, 70 and 80% of the power clean 1-RM. Data calculated as the mean across all subjects (peak: *n* = 22; average *n* = 18).

Individualized linear regression equations were calculated based upon four (30, 45, 70 and 80% of 1-RM; Figure 1) points using a custom-designed Microsoft Excel spreadsheet. 

Validity of the predicted 1-RMs with reference to the actual 1-RM was assessed via intraclass correlation coefficient (ICC), the standardized and raw typical error (TE), the coefficient of variation (CV) and effect size magnitudes (Cohen’s *d*). Associated 90% confidence limits (CL) were also calculated. The smallest worthwhile change (SWC) was set as 0.20 × the between-subject standard deviation (SD). The strength of the ICC was classified as: high (>0.9), acceptable (0.8 to 0.9) and questionable (0.7–0.8) [23], while the *r* was classified via the following criteria: trivial (<0.1), small (0.1–0.3), moderate (0.3–0.5), high (0.5–0.7), very high (0.7–0.9) or practically perfect (>0.9) [24]. An acceptability cut-off for the CV was set at <15% and was considered high when <5% [25,26]. A moderate and large standardized TE was indicated by 0.3 and 0.6, respectively. Cohen’s *d* magnitudes were classified as: trivial (<0.20), small (0.20–0.50), moderate (0.51–0.80) or large (>0.80) [27]. Paired *t*-tests were performed to determine the presence of a difference between conditions with an alpha set at ≤ 0.05. All validity analyses were performed using open access, peer-reviewed Excel (Microsoft, Redmond, Washington, USA) spreadsheets attained from Sportsci.org.

## 3. Results

Table 1 presents the predicted and actual velocity scores for the percentages of 1-RM power clean tested. 

**Figure 2:**
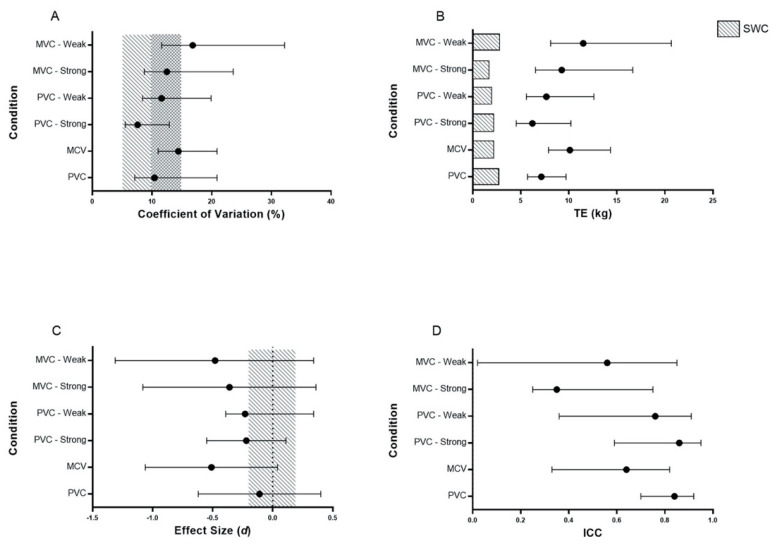
(**A**) Coefficient of variation (CV), (**B**) typical error (TE), (**C**) Cohen’s *d* effect size, and (**D**) intraclass correlation (ICC) of the four points predicted with respect to actual 1-RM power clean, alongside the 90% confidence intervals. The light and dark shaded regions in A represent good (5–10%) and acceptable (10–15%) validity, respectively. The shaded blocks in panel B indicate the smallest worthwhile change (SWC). A trivial effect size difference between the two conditions is indicated by the shaded area in panel C. PV-C: Peak velocity condition; MV-C: Mean velocity condition.

Figure 2 provides a graphical representation of the CV, TE, ICC and Cohen’s *d* across all conditions with respect to the criterion based upon four and three points, respectively. 

Table 2 and Table 3 present the measures of validity across all conditions with respect to the actual 1-RM. 

Across the entire sample, the ICC indicated acceptable validity for the PV-C (ICC = 0.86, 90% CI = 0.73–0.93), however, acceptability was not reached for the MV-C (ICC = 0.64, 90% CI = 0.33–0.82). When the predicted 1-RM was compared to the criterion (actual 1-RM) across the entire cohort, a large standardized TE was present for both experimental conditions (PV-C: standardized TE = 0.64, 90% CI = 0.42 to 1.03; MV-C: standardized TE = 1.29, 90% CI = 0.71–3.40). An acceptable CV was revealed for the PV-C (CV = 10.4, 90% CI = 7.1 to 20.9%), representative of a raw TE of 7.15 kg (90% CI = 5.71–9.71 kg). Acceptability was also reached within the MV-C, albeit to a reduced extent (CV = 14.4% (90% CI = 11 to 21%)), representative of a raw TE of 10.12 kg (90% CL = 7.89 to 14.34)). For both PV-C and MV-C, the raw TE exceeded the SWC (PV-C: 2.60 kg (90% CL = 2.08 to 3.54 kg), MV-C: 2.49 kg (90% CL = 1.94 to 3.52 kg)). Very high (r = 0.84 (90% CL = 0.70 to 0.92)) and high (r = 0.61 (90% CL = 0.28 to 0.81)) correlations were present for PV-C and MV-C, respectively, with reference to the criterion condition. The PV-C and MV-C displayed a trivial (Cohen’s *d*: −0.11 (90% CL = −0.62 to 0.40)) and moderate (Cohen’s *d*: −0.51 (90% CL = −1.06 to 0.04)) difference, respectively, in comparison to the criterion. A significant difference was found between the MV-C, but not the PV-C, and the criterion measure (MV-C: *p* = 0.37; PV-C: *p* = 0.02). When stratified on strength level, the stronger subjects displayed a greater degree of validity, as indicated by the ICC (stronger: 0.86 (90% CL = 0.59 to 0.95); weaker: 0.76 (90% CL = 0.36 to 0.91)) in the PV-C, but not the MV-C (stronger: 0.35 (90% CL = 0.25 to 0.75); weaker: 0.56 (90% CL = 0.02 to 0.85)). CV, TE and r measures by strength level are presented in Figure 2. Additionally, the stronger subjects’ 1-RM displayed a small non-statistically significant lower PV (1.74 ± 0.13 m·s^−1^, *p* = 0.34, ES = −0.43) when compared to the weaker subjects (1.83 ± 0.26 m·s^−1^).

## 4. Discussion

The primary aim of the current investigation was to determine if the load-velocity relationship could be used with a linear regression analysis to predict the 1-RM in the power clean. Based upon the results of the present study, the 1-RM power clean can be accurately estimated from a load-PV profile, whilst the load-MV profile does not allow for the accurate prediction of the power clean 1-RM. Additionally, the overall ability of the load-velocity relationship to accurately predict the power clean 1-RM increases with the overall strength of the individual, while the ability of the load-velocity relationship to detect the SWC in performance is considerably limited. 

Conceptually, the linear and negative relationship between the load lifted and the velocity of movement can be used to estimate the 1-RM for a resistance training exercise [28]. Based upon this relationship, several authors have suggested that the 1-RM can be accurately estimated with a variety of exercises including the bench press [10,11], bench-pull [11], seated military press [9] and back squat [2,12]. Careful inspection of the literature reveals that the majority of the research that supports the utility of the load-velocity relationship to estimate the 1-RM has been examined with the use of resistance training exercises performed in a Smith machine [9,10,11,12]. When free-weight resistance training exercises (e.g., deadlift and back squat) have been examined, the ability of the load-velocity relationship to estimate the 1-RM is less convincing [7,13,14]. One possible explanation for the reduced 1-RM prediction accuracy with free-weight exercises may be related to the fact that these exercises have a relevant amount of movement outside of the vertical plane. While the GYM linear position transducer directly measures vertical displacement, horizontal movement is quantified using basic trigonometry, which can increase the likelihood of measurement error [21]. As such, it is possible that as the potential for horizontal movement increases, there is a concomitant decrease in the ability to accurately predict a 1-RM from a load-velocity profile. 

Weightlifting exercises and derivatives are commonly utilized in the strength and conditioning environment due to their ability to develop key attributes which underpin sports performance [29]. Careful examination of weightlifting movements, such as the power clean and power snatch, reveal that they have horizontal and vertical movement patterns which are related to the engagement of the double knee bend technique [30] and how the barbell is caught. Due to the fact that these exercises have a large degree of horizontal movement, the ability to create a load-velocity profile that accurately predicts the 1-RM may be questioned. Conversely, the fact that a linear relationship exists between the load lifted and the velocity of movement during weightlifting exercises suggests that a load-velocity profile could in fact be constructed whilst using a linear position transducer during the power clean exercise [31,32,33,34]. For example, Cormie et al. [31] present data that the PV achieved during a power clean displays a linear reduction that corresponds to an increase in load from 30 to 90% of 1-RM (i.e., 30, 40, 50, 60, 70, 80 and 90%). Additionally, Marriner et al. [33] also report that there is a linear decrease in velocity when power clean loads are increased from 50 to 90% of 1-RM (i.e., 50, 70 and 90%). To the authors’ knowledge, only two studies have presented load-velocity data that include the velocity at 1-RM as part of the load-velocity profile for the power clean [32,34]. For example, Oranchuk et al. [32] and Ncalerio and Larumbe-Zabala [34] report that as the load lifted in the power clean increases toward the 1-RM, there is a linear reduction in the PV of movement, moving from a PV of 2.08 to 1.84 m·s^−1^. The present study also suggests that the velocity of movement decreases in a linear manner as the load lifted during the power clean increases (Figure 1). Similar peak velocities at the 1-RM in the power clean are noted between the present study and those published in the scientific literature [32,34]. In the present study, a PV at 1-RM of 1.79 ± 0.20 m·s^−1^ was achieved. These results are similar to the PV at 1-RM during the power clean of 1.60 ± 0.30 m·s^−1^ reported by Ncalerio and Larumbe-Zabala [34] and the PV of 1.81 ± 0.12 m·s^−1^ reported by Oranchuk et al. [32]. Based upon these data and previous research that suggests that the 1-RM can be estimated from the load-velocity profile, it is possible that the 1-RM in the power clean can be estimated from the load-velocity profile. 

While the MV is often recommended to be used when analyzing the load-velocity profile, it has been suggested that when looking at ballistic [35] or semi-ballistic movements, the use of PV may result in more accurate and reliable predictions of the 1-RM [36]. In the present study when the entire group was examined, there was no significant difference (*p* = 0.367, *d* = −0.11) between the predicted 1-RM (75.5 ± 12.8 kg) and the criterion measure (76.9 ± 13.0 kg) when PV was used in conjunction with a four-point linear regression analysis (Figure 1a). While both prediction equations resulted in predicted 1-RM values that were not significantly different than the criterion measure, these results need to be interpreted with caution. Firstly, the estimated 1-RM was less than the criterion measure when using either a four-point (−1.42 ± 7.21 kg) or three-point (−3.63 ± 9.78 kg) prediction. Secondly, it is important to note that the error of prediction exceeds the SWC for both the four-point (TE = 7.15; SWC = 2.60) and the three-point (TE = 8.02; SWC = 2.60) predictions. Another important factor to consider is that while the ICC was acceptable for the four-point prediction (ICC = 0.86), the lower bound was below the 0.80 threshold set for this investigation. Conversely, when looking at the three-point prediction, the ICC failed to reach the acceptable level (ICC = 0.78). Finally, while both the four-point (CV = 10.4%) and three-point prediction (CV = 12.8%) reached an acceptable level, the upper bound exceed the 15% boundary set of the present study. Due to these issues, the use of either prediction method with the use of the PV contains too much noise to properly use it as a tool to monitor meaningful changes in power clean strength. 

While some researchers suggest that PV is a more appropriate measure when examining ballistic movements [36,37], like the power clean, an alternative approach is to construct the load-velocity profile using the mean velocity [38]. García-Ramos et al. [38] have presented data that suggest that MV is the most appropriate variable to monitor during ballistic exercises, such as the bench press throw, performed in a Smith machine. Conversely, Jukic et al. [39] have recently reported that when the MV is used as part of a five-point linear equation, the deadlift 1-RM is overestimated by 7.3 ± 6.6 kg when compared to the actual 1-RM. In the present study when a four-point linear regression analysis was used, the predicted 1-RM (69.4 ± 12.9 kg) was significantly less (−6.59 ± 11.13 kg, *p* = 0.023, *d =* −0.52) than the criterion measure (76.5 ± 12.4 kg). Additionally, when a three-point linear regression analysis was used, the predicted 1-RM (66.4 ± 14.5 kg) was significantly less (−10.12 ± 14.29 kg, *p* = 0.008, *d* = −0.75) than the criterion measure. The inability to accurately predict the 1-RM from the load-MV profile in the present study agrees with existing research looking at multi-joint free-weight exercises such as the back squat [7] and deadlift [13,14]. Banyard et al. [7] report that when the mean concentric velocity during the back squat is used as part of the load-velocity relationship, the predicted 1-RM is generally overestimated. Similarly, Ruf et al. [13] report that the predicted 1-RM is generally overestimated by 5–10 kg in the deadlift when MV is used as part of the load-velocity analysis. In addition, it is important to note that the error of prediction exceeds the SWC for both the four-point (TE = 10.12; SWC = 2.60) and three-point predictions (TE = 8.02; SWC = 2.60). When examining the ICC, neither the four-point (ICC = 0.64) nor three-point (ICC = 0.42) predictions meet the 0.80 threshold set for this investigation. Finally, while the four-point prediction meets the minimum requirement for the CV (CV = 14.40), the upper boundary does exceed the 15% boundary set of the present study. Conversely, the three-point prediction does not meet the acceptable level of CV established for this study. Based upon these issues, MV is unable to be used as a method for monitoring changes in power clean strength due to the excessive noise contained in the data set. 

Studies comparing 1-RM velocities in a weightlifting derivative between stronger and weaker individuals are scarce. However, James et al. [26] reported the 1-RM power clean PV to be practically lower in those with greater strength in the lift (stronger: 1.72±0.06 m/s; weaker: 1.90±0.27 m/s; PV: *d* = −0.89, 95%CI = −1.87 to 0.10). Similar findings have also been reported when examining other traditional resistance training exercises, such as the bench press [40]. Recently, Ormsbee et al. [40] reported that the velocity at 1-RM for stronger people (0.14 ± 0.4 m·s^−1^) was significantly slower than those demonstrated for weaker individuals (0.20 ± 0.5 m·s^−1^). These previous findings are in alignment with the present study, where PV at failure during the power clean for the stronger group was practically lower (1.74 ± 0.13 m·s^−1^, *p* = 0.34, ES = −0.43) than the weaker group (1.83 ± 0.26 m·s^−1^). In the case of the power clean, the reduced velocity (and consequently displacement) required suggests that other factors (i.e., technical proficiency, timing of force application) play a greater role in power clean performance as strength level in this lift increases. Based upon these data, it is possible that as subjects become more trained and increase their overall maximal strength, their PV at 1-RM and at submaximal loads will be more reliable and thus it is possible that the estimates of 1-RM from PV will also become more reliable. Further research is warranted to explore the impact of increasing maximal strength levels on both the reliability and accuracy of 1-RM estimates from PV.

While the present study adds to our current understanding of the utility of the load-velocity profile and its ability to either monitor performance change or estimate the 1-RM in the power clean, the present study does have limitations. Firstly, while the number of subjects utilized in the present study is similar to other studies [7,13,14] that have examined the load-velocity profile in multi-joint ground-based free-weight exercises, studies with larger samples sizes are warranted. 

Secondly, the study required the load-velocity profile to be determined four to seven days after the 1-RM test. While it is possible that the predictive ability of the load-velocity profile was impacted by the time course between the maximum test and the development of the load-velocity profile, there is evidence to suggest that the power clean 1-RM is relatively stable during this time frame [41]. For example, Comfort and McMahon [41] have reported that the power clean 1-RM is reliable and varies by <1.36 kg when re-tested three to five days after an initial 1-RM test. Therefore, it is likely that the time between the 1-RM and the development of the load-velocity profile exerted a relatively minimal impact on the outcome measures of the present study. Even though it is likely that there is a minimum impact from the method used in the present study, further research is warranted to replicate the methods of Banyard et al. [7], who tested the 1-RM and load-velocity profile every other day for one week in order to truly understand the reliability of the 1-RM power clean and the load-velocity profile. 

## 5. Conclusions

The primary goal of the present investigation was to determine if PV or MV could be used with a linear regression to predict the power clean 1-RM. Based upon the findings of the present study, it appears that the power clean 1-RM may be estimated from a load-PV profile. However, it is important to note that there is excessive noise in this estimate, which may reduce the capacity of this measure as a monitoring tool. Due to the lack of ability to produce reliable estimates, as demonstrated by the large TE, strength and conditioning professionals should be cautious when using these estimates to monitor changes in 1-RM power clean. If strength and conditioning professionals decide to use the load-velocity profile to estimate the power clean 1-RM, they should create a four-point (30%, 45%, 70% and 80% of 1-RM) load-velocity profile utilizing the PV as part of the linear regression analysis. It is not recommended to create load-velocity profiles with the MV when trying to estimate the 1-RM power clean.

## Figures and Tables

**Table 1:** Predicted and actual velocity scores (±90% confidence intervals) for percentages of the one-repetition maximum power clean.

**Peak Velocity (m·s^−1^)**				
**%1-RM**	**Actual** **(90% CI)**	**Predicted** **(90% CI)**	***p***	**d**
30	3.29 (3.07–3.51)	3.37 (3.34–3.41)	0.48	0.21
45	2.82 (2.68–2.96)	3.13 (3.07–3.18)	<0.01	1.01
70	2.32 (2.22–2.43)	2.69 (2.60–2.78)	<0.01	1.19
80	2.18 (2.07–2.29)	2.52 (2.42–2.61)	<0.01	1.13
100	1.79 (1.71–1.87)	2.20 (2.09–2.32)	<0.01	1.28
**Mean Velocity (m·s^−1^)**				
**%1-RM**	**Actual** **(90% CI)**	**Predicted** **(90% CI)**	***p***	**d**
30	1.89 (1.77–2.00)	1.95 (1.92–1.97)	0.38	0.32
45	1.54 (1.42–1.67)	1.78 (1.74–1.81)	<0.01	1.00
70	1.22 (1.12–1.32)	1.47 (1.41–1.53)	<0.01	1.17
80	1.09 (1.00–1.18)	1.35 (1.29–1.41)	<0.01	1.18
100	0.92 (0.84–1.00)	1.13 (1.04–1.21)	<0.01	0.98

**Table 2:** Measures of validity using four incremental loads with respect to the actual one-repetition maximum attained.

	Four Point								
		TE STD	TE Raw (kg)	CV% (90% CI)	SWC (kg)	r	ES (90%CI)	ICC (90%CI)	*p*
Entire Group	Peak Velocity	0.64	7.15	10.4 (7.1–20.9)	2.60	0.84	−0.11 (−0.62–0.40)	0.86 (0.73–0.93)	0.367
	Mean Velocity	1.29	10.12	14.4 (11.0–20.9)	2.49	0.61	−0.51 (−1.06–0.04)	0.64 (0.33–0.82)	0.022
Stronger	Peak Velocity	0.66	6.21	7.6 (5.5–12.9)	2.49	0.84	−0.22 (−0.55–0.11)	0.86 (0.59–0.95)	0.262
Weaker	Peak Velocity	0.80	7.67	11.6 (8.4–19.9)	2.32	0.78	−0.23 (−0.39–0.34)	0.76 (0.36–0.91)	0.916
Stronger	Mean Velocity	3.13	9.27	12.5 (8.7–23.6)	1.82	0.3	−0.36 (−1.08–0.36)	0.35 (0.25–0.75)	0.391
Weaker	Mean Velocity	1.53	11.50	16.8 (11.6–32.2)	2.57	0.55	−0.48 (−1.31–0.34)	0.56 (0.02–0.85)	0.026

Note: TE: Typical error; STD: Standardized; CV: Coefficient of variation; SWC: Smallest worthwhile change; r: Pearson’s correlation; ES: Cohen’s *d* effect size; CI: Confidence interval.

**Table 3:** Measures of validity using three incremental loads with respect to the actual one-repetition maximum attained.

	Three Point								
		TE STD	TE Raw (kg)	CV% (90% CI)	SWC (kg)	r	ES (90%CI)	ICC (90%CI)	*p*
Entire Group	Peak Velocity	0.75	8.02	12.8 (8.6–25.9)	2.60	0.80	−0.25 (−0.49–0.00)	0.78 (0.58–0.89)	0.097
	Mean Velocity	2.01	11.48	15.1 (10.2–31.0)	2.49	0.44	−0.71 (−1.13–−0.28)	0.42 (−0.01–0.70)	0.008
Stronger	Peak Velocity	0.67	6.32	7.5 (4.8–18.6)	2.15	0.83	−0.32 (−1.07– 0.43)	0.78 (0.47–0.92)	0.470
Weaker	Peak Velocity	0.99	8.60	13.3 (8.5–34.6)	2.32	0.71	−0.22 (−0.97–0.53)	0.89 (0.7–0.96)	0.620
Stronger	Mean Velocity	1.79	8.49	3.8 (2.4–9.2)	1.82	0.49	−0.75 (−1.53–0.04)	0.55 (−0.02–0.85)	0.110
Weaker	Mean Velocity	16.08	13.72	16.9 (10.7–44.9)	2.57	0.06	−0.88 (−1.64–−0.13)	−0.10 (−0.50–0.60)	0.060

Note: TE: Typical error; STD: Standardized; CV: Coefficient of variation; SWC: Smallest worthwhile change; r: Pearson’s correlation; ES: Cohen’s *d* effect size; CI: Confidence interval.
